# Immune Cell-Type Specific Ablation of Adapter Protein ADAP Differentially Modulates EAE

**DOI:** 10.3389/fimmu.2019.02343

**Published:** 2019-10-01

**Authors:** Jochen Rudolph, Clara Meinke, Martin Voss, Karina Guttek, Stefanie Kliche, Dirk Reinhold, Burkhart Schraven, Annegret Reinhold

**Affiliations:** ^1^Institute for Molecular and Clinical Immunology, Otto von Guericke University Magdeburg, Magdeburg, Germany; ^2^Health Campus Immunology, Infectiology and Inflammation, Magdeburg, Germany

**Keywords:** adapter protein, ADAP, EAE, conditional knockout mice, T cell, NK cell, myeloid cell, platelet

## Abstract

The cytosolic adhesion and degranulation-promoting adapter protein ADAP is expressed in various hematopoietic cells including T cells, NK cells, myeloid cells, and platelets but absent in mature B cells. The role of ADAP in T cell activation, proliferation and integrin activation is well-accepted. We previously demonstrated that conventional ADAP knockout mice show a significantly attenuated course of experimental autoimmune encephalomyelitis (EAE). To dissect the impact of different ADAP expressing cell populations on the reduced EAE severity, here, we generated lineage-specific conditional knockout mice. ADAP was deleted in T cells, myeloid cells, NK cells and platelets, respectively. Specific loss of ADAP was confirmed on the protein level. Detailed immunophenotyping was performed to assess the consequence of deletion of ADAP with regard to the maturation and distribution of immune cells in primary and secondary lymphoid organs. The analysis showed equivalent results as for conventional ADAP knockout mice: impaired thymocyte development in ADAP^fl/fl^ Lck-Cre mice, normal NK cell and myeloid cell distribution in ADAP^fl/fl^ NKp46-Cre mice and ADAP^fl/fl^ LysM-Cre mice, respectively as well as thrombocytopenia in ADAP^fl/fl^ PF4-Cre mice. Active EAE was induced in these animals by immunization with the myelin oligodendrocyte glycoprotein_35−55_ peptide. The clinical course of EAE was significantly milder in mice with loss of ADAP in T cells, myeloid cells and NK cells compared to ADAP-sufficient control littermates. Surprisingly, specific deletion of ADAP in platelets resulted in a more exacerbated disease. These data show that T cell-independent as well as T cell-dependent mechanisms are responsible for the complex phenotype observed in conventional ADAP knockout mice.

## Introduction

Adapter proteins contain modular domains that mediate constitutive or inducible interactions between proteins or proteins and lipids. By definition, they exhibit no enzymatic or transcriptional activity. The adhesion and degranulation-promoting adapter protein ADAP belongs to the group of cytosolic adapter proteins. ADAP is expressed within the hematopoietic system in T cells, NK cells, myeloid cells and platelets, but not in mature B cells (1, 2).

The function of ADAP is well-described in T cells. ADAP was the first adapter protein identified that is involved in inside-out signaling linking the TCR-stimulation to integrin activation (3, 4). ADAP-deficient mice show impaired thymic development, reduced TCR-induced integrin-dependent adhesion, decreased proliferation as well as diminished NF-κB activation and cytokine production (5–7). Interestingly, the loss of ADAP showed strong impact on CD4^+^ T cell activation, expansion and effector function, whereas CD8^+^ T cells revealed only moderate effects of ADAP deficiency (8). At the same time, Fiege et al. (9) provided evidence for a negative regulatory role of ADAP in the conversion of naïve CD8^+^ T cells into memory phenotype under steady state condition. Furthermore, ADAP is a positive regulator for resident CD8^+^ T cell memory formation during an acute pathogen infection (10). These data suggest that ADAP fulfills different function in CD4^+^ and CD8^+^ T cells.

In NK cells, ADAP is exclusively responsible for cytokine and chemokine secretion but not for cytotoxicity (11, 12). Neutrophils from ADAP-deficient mice show defective E-selectin-mediated integrin activation and slow leukocyte rolling in the mouse kidney ischemia-reperfusion model (13). The role of ADAP in other myeloid cells like mast cells, monocytes and macrophages remains largely unknown. ADAP-deficiency in microglia, the CNS-resident macrophage population does not influence microglia function such as NO production and cytokine release (14). Limited data are available about the role of ADAP in dendritic cells (DC). Previous results revealed that ADAP is critically involved in CD11c integrin-mediated cytokine production and actin polymerization (15).

In addition to lymphoid and myeloid cells, ADAP is required for selected platelet functions. ADAP-deficient mice show mild thrombocytopenia, normal bleeding time but more frequent re-bleeding from tail wounds (16). Moreover, ADAP-deficient platelets form unstable thrombi after carotoid artery occlusion (17). It was clearly demonstrated that ADAP interacts with the integrin binding proteins talin and kindlin-3. ADAP-deficient platelets exhibit decreased association with talin and kindlin-3 leading to reduced activation of integrin αIIbβ3 and decreased fibrinogen binding (18). In addition, ADAP is also involved in the regulation of platelet integrin α2β1 (19).

Taken together, independent of the cell type, the adapter protein ADAP is implicated in cell functions associated with integrin activation, cytoskeletal rearrangement and adhesion. Therefore, it is tempting to speculate that the loss of ADAP has an impact on immune-mediated diseases. We previously investigated the clinical course of experimental autoimmune encephalomyelitis (EAE), the most commonly used mouse model of the human disease multiple sclerosis, in conventional ADAP knockout mice. Indeed, we found that ADAP-deficient mice show significantly milder EAE compared to ADAP-sufficient wildtype mice (20).

EAE is a CNS demyelinating disease caused by autoreactive T cells directed against myelin proteins like MOG (myelin oligodendrocyte glycoprotein). The disease can be induced in susceptible animals by immunization with myelin peptides in combination with strong immune adjuvants like complete Freund's adjuvant. In addition, most protocols for induction of EAE in rodents use pertussis toxin to block G-protein coupled receptors and to enhance immune response (21). The histopathology consists of meningeal and perivascular inflammation dominated by activated CD4^+^ T cells and macrophages and enhanced microglia reactivity (22). This pathological process leads to axonal injury and the formation of demyelinated plaques. In C57BL/6 mice, the disease shows a monophasic chronic-progressive clinical profile. The typical symptoms of diseased mice vary from tail plegia followed by hind limb and forelimb paralysis (21).

It is well-accepted that EAE is primarily driven by encephalitogenic helper T cells (Th1 and Th17) and regulatory T cells (23). During the induction phase of the disease, myelin-specific CD4^+^ T cells are activated and expand in the peripheral lymphoid tissue. These effector T cells cross the blood-brain barrier and enter the CNS. The inflammatory response leads to the recruitment of other immune cells including monocytes, macrophages, dendritic cells, B cells, and NK cells (24). The invading monocytes, macrophages and dendritic cells express high amounts of MHC-II molecules and are involved in antigen presentation and reactivation of T cells within the CNS. In addition, monocytes and macrophages secrete pro- as well as anti-inflammatory cytokines depending on their environment (25). Also CD8^+^ T cells are involved in cytotoxicity, pro-inflammatory cytokine production and demyelination during disease induction as well as in regulatory function during down modulation of inflammation (26). The role of NK cells in EAE is still controversial. NK cells contribute to both effector function via their cytotoxic activity and to regulatory function via the production of pro- and anti-inflammatory cytokines. It has been reported that NK cell depletion results in more exacerbated disease (27). The importance of NK cell-derived IFN-γ for macrophage expansion during early phase of EAE was clearly demonstrated (28). Furthermore, it was shown that platelets contribute to the pathogenesis of EAE by promoting the inflammatory response in the CNS. Depletion of platelets during the effector phase of EAE significantly ameliorated the disease progression (29).

All above-mentioned hematopoietic cells—T cells, NK cells, myeloid cells, and platelets - express the adaptor protein ADAP. We have previously demonstrated that conventional ADAP knockout mice show strongly attenuated EAE. However, this approach cannot answer the question, which cells contribute to the lower EAE severity. To dissect the role of ADAP in different cell types during EAE, we generated conditional knockout mice. After characterization of the immune system of these mice, we induced active EAE by application of MOG_35−55_ peptide in complete Freund's adjuvant (CFA) and monitored the disease course.

## Materials and Methods

### Mice

Mice containing the knockout first allele C57BL/6N-Fyb^tm1a(EUCOMM)Hmgu^/Cnrm (30) were sourced from the EUCOMM project and were purchased from the European Mouse Mutant Archive EMMA. The lacZ and neomycin-resistance cassettes were both removed by breeding with transgenic mice expressing an Flp recombinase resulting in floxed alleles (containing *loxP* sites) and restoring the wildtype. To generate mice with the deletion of ADAP in a specific cell lineage, mice with floxed alleles were crossed with mice carrying the Cre recombinase. To delete ADAP in the megakaryocytic lineage, the Cre recombinase was under control of the platelet factor-4 (PF4) promotor as previously described (31). To delete ADAP in thymocytes and T cells, the B6.Cg-^Tg(Lck−cre)548Jxm^/J mouse strain expressing the Cre recombinase under control of the lymphocyte protein tyrosine kinase (Lck) promotor was provided by Prof. Ursula Bommhardt (Magdeburg). To generate mice with the deletion of ADAP in the NK cell lineage, the NKp46-iCre knock-in mice were provided by Prof. Eric Vivier (Paris) (32). To delete ADAP in the myeloid cell lineage, we used the LysM-Cre knock-in mice, where the Cre recombinase was inserted into the lysosome 2 gene (B6N.129P2(B6)-Lyz2 ^tm1(cre)Ifo^/J, provided by Prof. Peter Mertens, Magdeburg).

The general scheme of generation of conditional ADAP knockout mice is shown in Figure S1. The presence or absence of the *FRT* sites, the *loxP* sites, the gene of interest and the respective Cre transgene were checked routinely by PCR using genomic DNA isolated from ear tissue. The primer sequences are listed as Table S1.

Conventional ADAP-deficient mice (6) were backcrossed to C57BL/6JBom for at least ten generations. For all experiments, 8–14 week old animals were used. To investigate specific effects of ADAP deletion and to exclude off target effects of Cre recombinase, ADAP^wt/wt^ Cre^tg^ (Cre control) and ADAP^fl/fl^ Cre^tg^ (conditional k.o.) mice were always used as littermates.

Animals were bred and maintained under specific-pathogen-free conditions in the central animal facility of the medical faculty of the University of Magdeburg. All procedures were conducted according to protocols approved by the local authorities (Landesverwaltungsamt Sachsen-Anhalt; reference number: 42502-2-1273 UniMD).

### EAE Induction

Induction of EAE was performed as described earlier (33). Briefly, active EAE was induced by immunization with 200 μg MOG_35−55_ peptide emulsified in complete Freund's adjuvant (CFA, Sigma-Aldrich) containing 800 μg of heat-killed *Mycobacterium tuberculosis* (Difco Laboratories). The emulsion was administered s.c. as four 50-μl injections into the flanks of each leg. In addition, 200 ng of pertussis toxin (List Biological Laboratories) dissolved in 200 μl PBS was injected i.p. on days 0 and 2 after immunization as described earlier (34). Mice were monitored daily for clinical signs of EAE and graded on a scale of increasing severity from 0 to 5 as follows: 0, no signs; 0.5, partial tail weakness; 1, limp tail or slight slowing of righting from supine position; 1.5, limp tail and slight slowing of righting; 2, partial hind limb weakness or marked slowing of righting; 2.5, dragging of hind limb(s) without complete paralysis; 3, complete paralysis of at least one hind limb; 3.5, hind limb paralysis and slight weakness of forelimbs; 4, severe forelimb weakness; 5, moribund or dead (35). For reasons of animal welfare, mice were killed when reaching a score of 3 or above. Mean clinical scores at each day were calculated by adding disease scores of individual mice divided by the number of mice in each group.

### Flow Cytometry

To investigate the distribution of immune cells, cells isolated from thymus, bone marrow, spleen and peripheral blood were stained with the following antibodies: anti-CD3ε (clone 145-2C11), anti-CD4 (clone RM4-5), anti-CD8 (clone 53-6.7), anti-CD11b, anti-CD11c (clone N418), anti-NKp46 (clone 29A1.4), anti-NK1.1 (clone PK136), anti-B220 (clone RA3-6B2), anti-IgM (clone RMM-1), anti-IgD (clone 11-26c2a), and the respective isotype controls were purchased from BioLegend. Flow cytometric measurements were performed as 4-color analysis using FACSCalibur and Cellquest software (BD Biosciences).

For the detection of NK cell development in the bone marrow, cells were treated with Fc block (purified anti-CD32/CD16 monoclonal antibodies) and subsequently stained with anti-CD3ε-FITC, anti-CD4-FITC, anti-CD8-FITC, anti-CD19-FITC (clone 6D5), anti-Gr-1-FITC (clone RB6-8C5), anti-NKp46-BV450 (BD Biosciences), anti-NK1.1-PacificBlue, and anti-CD122-PE-Cy7 (clone T41-B1), anti-CD11b-APC/Cy7 (cloneM1/70). For detection of myeloid cell distribution, in addition anti-F4/80 (clone BM8), anti-CD11c (clone N418) and anti-Ly6G (clone 1A8) were used. Antibodies were purchased from BioLegend if not otherwise indicated. All steps were performed on ice and incubations at 4°C in the dark. After staining, cells were fixed with 2% PFA in PBS.

For intracellular ADAP staining, we used a polyclonal sheep anti-ADAP antiserum and the respective pre-immune serum kindly provided by G. Koretzky. The staining procedure was previously described (1) and works reliably to track ADAP protein expression. Briefly, the cells were first stained with the fixable viability dye eFluor® 506 (eBioscience) and then staining of cell surface molecules and fixation (2% paraformaldehyde) were performed. Fixed cells were permeabilized in Hank's Salt Solution (HSS) containing 2% FCS and 0.5% Saponin and intracellular staining with antisera and secondary donkey anti-sheep-FITC was performed in HSS/2% FCS/0.2% Saponin. These measurements were performed using a BD LSRFortessa and BD FACSDiva™ software (BD Biosciences) and analyzed with FlowJo® v10. Analyses were performed on viable and single cells.

For detection leukocytes in the brain, mice were euthanized by inhalation of CO_2_. The abdominal cavity and thorax were opened immediately and transcardial perfusion was performed through the left ventricle using NaCl. The brain was carefully removed, cut into pieces and treated with collagenase (2.5 mg/ml, Roche Diagnostics) and DNAse I (1 mg/ml, SIGMA) for 45 min at 37°C. Tissue was ground through a cell strainer (70 μm), washed, resuspended in 37% Percoll, and layered onto 70% Percoll. After centrifugation (600 g, 25 min), cells were removed from the interphase, washed and stained for FACS analysis.

### TGF-β1 Measurement

Platelet-poor plasma was obtained by double centrifugation (400 × g for 5 min and subsequently 6.000 × g for 15 min). Plasma concentrations of murine latent TGF-β1 were measured according to the instructions of the manufacturer using a specific ELISA kit (R&D Systems).

### Statistical Analysis

Results are expressed as mean ± SEM. Unpaired Student's *t*-test was used to assess the statistical significance of the differences. Statistical comparison of EAE disease severity between different two groups of animals was accomplished by performing non-parametric Wilcoxon matched pairs test using GraphPad Prism software. *P* < 0.05 (^*^); *P* < 0.01 (^**^); *P* < 0.001 (^***^).

## Results

### T Cell-Specific Conditional ADAP Knockout Shows Less Severe EAE

**Figure 1:**
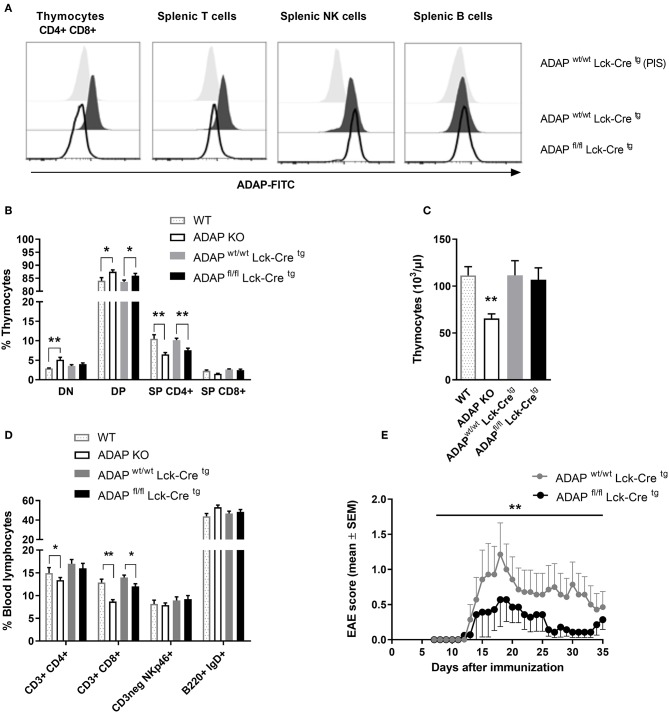
Characterization of mice with specific deletion of ADAP in T cells. **(A)** Anti-ADAP serum or preimmune serum (PIS) were used to detect ADAP protein levels by flow cytometry in double positive CD4^+^ CD8^+^ thymocytes and in T cells (CD3^+^), NK cells (CD3neg, NK1.1^+^), and B cells (CD19^+^) in the spleen from conditional knockout mice and Cre control mice. Representative histograms are results from three independent experiments. **(B)** Thymocytes were isolated from conditional knockout mice (ADAP^fl/fl^ Lck-Cre^tg^), Cre control mice (ADAP^wt/wt^ Lck-Cre^tg^), conventional ADAP knockout mice (ADAP KO), and respective wildtype control mice (WT). The frequencies of CD4^−^ CD8^−^ double negative thymocytes (DN), CD4^+^ CD8^+^ double positive thymocytes (DP), and single positive thymocytes (SP CD4^+^, SP CD8^+^) were analyzed by flow cytometry. **(C)** Absolute numbers of thymocytes per animal from conditional knockout mice, Cre control mice and conventional ADAP knockout mice are given. **(D)** The lymphocyte subpopulations in the peripheral blood were stained with the indicated antibodies. All results **(B–D)** are expressed as means + SEM of data from six independent experiments (*P < 0.05; **P < 0.01). **(E)** Active EAE was induced following immunization with MOG_35−55_ peptide in CFA at day 0 in combination with pertussis toxin at day 0 and day 2. The clinical score of active EAE was assessed for 35 days after immunization. Data are shown as means ± SEM [*n* = 8 animals per group; **P < 0.001 (conditional ADAP knockout vs. Cre control)].

The phenotype of ADAP-deficient murine T cells is well-described (6). Therefore, we started to generate a T cell-specific ADAP knockout mouse using the commercially available C57BL/6N-Fyb^tm1a(EUCOMM)Hmgu^/Cnrm (EUCOMM) mouse (Figure S1). The mouse strain containing the critical exon 2 of *fyb* gene flanked by 2 loxP sites was crossed to a transgenic mouse expressing Cre recombinase under control of the mouse Lck promotor. This promotor is active during embryonic stages of thymic development and in mature T cells (36). The T cell-specific knockout was confirmed by flow cytometry based on the intracellular ADAP staining with a specific polyclonal antiserum and the respective preimmune serum. As depicted in Figure 1A, CD4^+^ CD8^+^ double positive cells, representing the majority of thymocytes, showed a clear loss of ADAP expression in conditional knockout mice (ADAP^fl/fl^ Lck-Cre^tg^) compared to Cre control mice (ADAP^wt/wt^ Lck-Cre^tg^). This specific loss of ADAP expression was also seen in single positive CD4^+^ or CD8^+^ thymocytes of T cell-specific ADAP knockout mice (data not shown). In the spleen, the ADAP knockout was confirmed in mature T cells of ADAP^fl/fl^ Lck-Cre^tg^ mice, whereas NK cells showed ADAP expression as strong as those derived from ADAP^wt/wt^ Lck-Cre^tg^ mice. Since mature B cells are known to lack ADAP expression (1), ADAP was not detectable in splenic B cells (Figure 1A). Summarizing, these results indicated a specific loss of ADAP in thymocytes and T cells.

Next, we analyzed the constitution of primary lymphoid organs (thymus) and the peripheral blood of ADAP^wt/wt^ Lck-Cre^tg^ (Cre control) and ADAP^fl/fl^ Lck-Cre^tg^ (conditional k.o.) mice. Because of the known T-cell phenotype of conventional ADAP knockout mice, these animals and respective wildtype controls were included in the analysis. The distribution of double negative (CD4^−^ CD8^−^), double positive (CD4^+^ CD8^+^), CD4^+^, and CD8^+^ single positive thymocytes in conditional ADAP knockout mice reflected the distribution in conventional ADAP knockout mice: the percentage of double positive thymocytes was significantly increased and the percentage of CD4^+^ thymocytes was significantly reduced (Figure 1B). The total numbers of cells in the thymus of were only slightly reduced in conditional k.o.mice compared to Cre control mice, whereas the conventional k.o.mice revealed significantly reduced cell numbers (Figure 1C). This observation indicates the possibility that other ADAP expressing cells influence thymic development. In the peripheral blood, conventional ADAP k.o. mice displayed reduced numbers of T cell subpopulations (CD3^+^ CD4^+^ and CD3^+^ CD8^+^). In contrast, in conditional k.o. mice (ADAP^fl/fl^ Lck-Cre^tg^) only the CD3^+^ CD8^+^ subpopulation showed significant reduction in the percentage of cells, whereas the CD3^+^ CD4^+^ T cell subpopulation was only slightly reduced (Figure 1D).

Thus, these data demonstrate that the T-cell specific conditional ADAP knockout (ADAP^fl/fl^ Lck-Cre^tg^) resembles the phenotype of the conventional ADAP knockout, namely the decreased portion of single positive thymocytes and mature T cells. However, this reduction is clearly less pronounced in the conditional knockout mice compared with conventional knockout mice.

Next, we investigated the clinical course of active experimental autoimmune encephalomyelitis (EAE) in the T-cell specific conditional ADAP knockout mice. EAE is a strongly T-cell dependent demyelinating disease of the central nervous system. We have previously shown that conventional ADAP knockout mice show less severe EAE compared to wildtype mice, however, most likely mainly due to T cell-independent mechanisms (20). Active EAE was induced by immunization of mice with MOG_35−55_ peptide. As shown in Figure 1E, we observed a statistically significant less severe clinical course of EAE in mice exclusively devoid of ADAP expression in T cells compared with Cre control mice. These results indicate that ADAP is involved in both, T-cell dependent and T-cell independent mechanisms of this autoimmune disease.

### NK Cell-Specific Conditional ADAP Knockout Reveals Reduced EAE Severity

**Figure 2:**
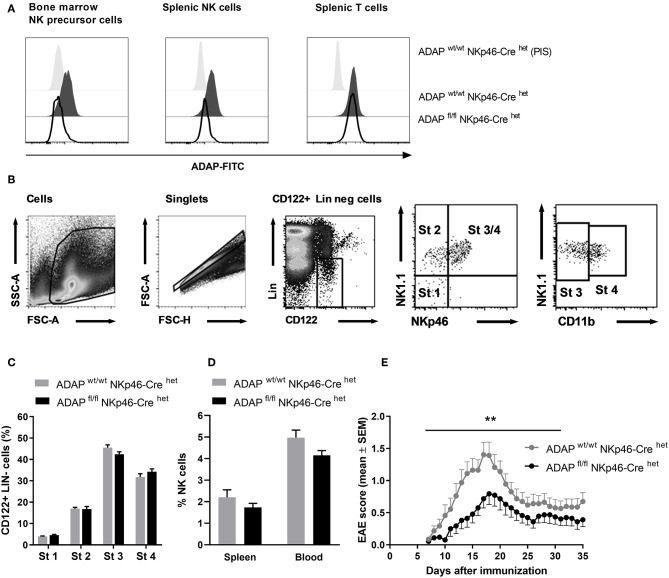
Characterization of mice with specific deletion of ADAP in NK cells. **(A)** Anti-ADAP serum or preimmune serum (PIS) were used to detect ADAP expression by flow cytometry in bone marrow NK cell precursors (left), in splenic NK cells (middle) and in splenic T cells (right) from conditional knockout mice (ADAP^fl/fl^ NKp46-Cre^het^) and Cre control mice (ADAP^wt/wt^ NKp46-Cre^het^). Histograms show representative results from three independent experiments. **(B)** The gating strategy for the analysis of NK cell development in the bone marrow is shown. The lineage staining excludes T cells (CD3^+^, CD4^+^, CD8^+^), B cells (CD19^+^) and granulocytes (Gr1^+^). The four different stages are depicted as dot plots according to their NK1.1, NKp46, and CD11b expression profile. **(C)** Quantification of the four different stages of NK cell development in the bone marrow is given as means + SEM of data from three independent experiments (*n* = 6 mice per group). **(D)** The graph shows frequencies of mature NK cells (CD3^−^, NK1.1^+^, NKp46^+^) in the spleen and in the peripheral blood from conditional knockout mice (ADAP^fl/fl^ NKp46-Cre^het^) and Cre control mice (ADAP^wt/wt^ NKp46-Cre^het^); mean values + SEM (*n* = 8 animals per group). **(E)** Active EAE was induced by immunization with MOG_35−55_ peptide in CFA at day 0 in combination with pertussis toxin at day 0 and day 2. The disease severity was recorded as clinical score for 35 days after immunization. Data are shown as means ± SEM (*n* = 23 animals per group from three independent experiments). **P < 0.001 conditional ADAP knockout vs. Cre control.

To delete ADAP specifically in NK cells, we made use of a “knock-in” mouse expressing the Cre recombinase under control of the NKp46 promotor. NKp46 is selectively expressed in NK cells with two exceptions reported: a minor T-cell subset and mucosal group-3 innate lymphoid cells (ILC3) (32). The NK-cell specific knockout was confirmed by intracellular ADAP staining as described. Bone marrow NK precursor cells of ADAP^wt/wt^ NKp46-Cre^het^ (Cre control) mice show expression of ADAP, whereas cells from ADAP^fl/fl^ NKp46-Cre^het^ (conditional k.o.) mice display loss of ADAP expression to the level of cells stained with preimmune serum (Figure 2A). The same holds true for the spleen: mature NK cells from Cre control mice express ADAP, whereas NK cells from conditional k.o. mice almost completely lost ADAP expression. In contrast, splenic T cells of ADAP^fl/fl^ NKp46-Cre^het^ (conditional k.o.) mice showed ADAP expression as strong as those derived from ADAP^wt/wt^ NKp46-Cre^het^ (Cre control) mice (Figure 2A).

We first investigated the different steps of NK cell maturation in the bone marrow (Figure 2B). NK cell differentiation is initiated at precursor stage (stage 1) that is characterized by the expression of CD122 (IL-2/IL-15 receptor ß chain) and the absence of lineage markers CD3 and CD19 (Figure 2B). Immature NK cells are defined by the appearance of NK1.1 (stage 2) followed by the expression of NKp46 (stage 3). Fully mature NK cells additionally acquire the expression of CD11b (stage 4). We analyzed whether the loss of ADAP in NK cells is associated with modifications in the NK cell differentiation. As depicted in Figure 2C, the percentage of NK cells at the different developmental stages in the bone marrow was comparable in conditional knockout mice (ADAP^fl/fl^ NKp46-Cre^het^) and Cre control mice (ADAP^wt/wt^ NKp46-Cre^het^). Thus, ADAP expression has no influence on NK cell differentiation. We next analyzed the constitution of secondary (spleen) lymphoid organs and peripheral blood of ADAP^wt/wt^ NKp46-Cre^het^ (Cre control) and ADAP^fl/fl^ NKp46-Cre^het^ (conditional k.o.) mice. As expected, the percentages of mature NK cells in spleen and blood were similar in mice of both genotypes (Figure 2D).

Besides encephalitogenic T cells, also NK cells are involved in the development of EAE by providing an early source of IFN-γ (28). ADAP-deficient NK cells from conventional k.o. mice revealed reduced IFN-γ secretion *in vitro* (11). Based on these data, we were curious about the clinical course of EAE in the NK-cell specific conditional ADAP knockout mice. As expected, the loss of ADAP expression exclusively in NK cells (ADAP^fl/fl^ NKp46-Cre^het^) caused a significantly less severe course of the disease compared to controls (Figure 2E).

### Deficiency of ADAP Restricted to Myeloid Cells Leads to Attenuated EAE Severity

**Figure 3:**
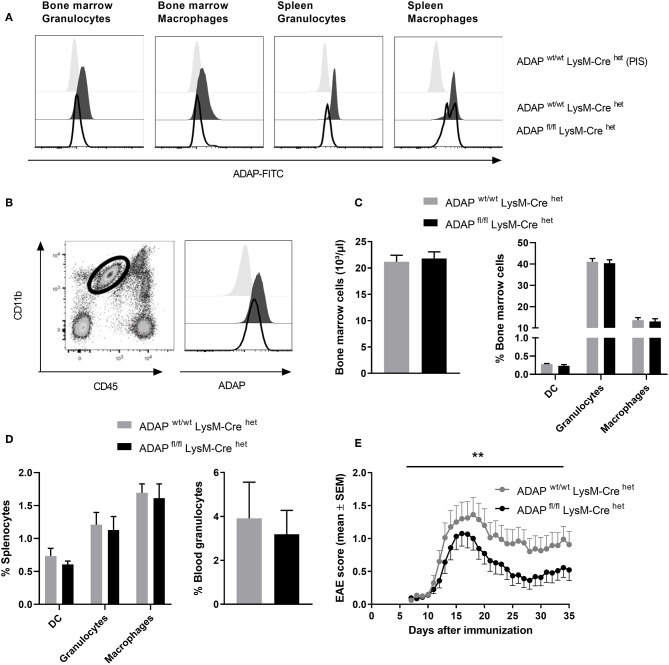
Characterization of mice with specific deletion of ADAP in myeloid cells. **(A)** Anti-ADAP serum or preimmune serum (PIS) were used to detect ADAP expression by flow cytometry in bone marrow granulocytes (CD11b^+^ Ly6G^+^) and bone marrow macrophages (CD11b^+^ F4/80^+^) as well as in granulocytes and macrophages from the spleen of conditional knockout mice (ADAP^fl/fl^ LysM-Cre^het^) and Cre control mice (ADAP^wt/wt^ LysM-Cre^het^). Histograms show representative results from three independent experiments. **(B)** Leukocytes were isolated from the brain by Percoll gradient, stained with antibodies against CD11b and CD45 and analyzed by flow cytometry. The region indicates the CD45 low, CD11b high brain-resident microglia (dot plot). The histograms show ADAP expression level in microglia cells from conditional knockout mice (ADAP^fl/fl^ LysM-Cre^het^; black line) and Cre control mice (ADAP^wt/wt^ LysM-Cre^het^; dark gray, filled). Cells stained with preimmune serum (light gray, filled) were used as control. The plots show representative results out of three independent experiments. **(C)** Frequencies of precursors of dendritic cells (CD11c^+^ DC), granulocytes (CD11b^+^ Ly6G^+^), and macrophages (CD11b^+^ F4/80^+^) in the bone marrow were determined using flow cytometry (left graph). The right graph illustrates the total number of bone marrow cells. **(D)** Distribution of mature dendritic cells, granulocytes and macrophages in the spleen (left graph) is shown together with the percentage of granulocytes in the peripheral blood (right graph). All results are expressed as means + SEM of data from minimum three independent experiments. **(E)** Active EAE was induced following immunization with MOG_35−55_ peptide in CFA. The clinical score of EAE was assessed for 35 days after immunization (means ± SEM; *n* = 20 animals per group from three independent experiments). **P < 0.01 conditional knockout mice (ADAP^fl/fl^ LysM-Cre^het^) vs. Cre control mice (ADAP^wt/wt^ LysM-Cre^het^).

The LysM-Cre knock-in mouse strain has a Cre recombinase inserted into the *lysozyme 2* gene. Cre-mediated recombination results in deletion of the floxed ADAP allele in the myeloid lineage including macrophages and neutrophils (37). Specific deletion was first monitored by PCR (Figure S1). On protein level, loss of ADAP in myeloid cells was confirmed by intracellular staining with the specific anti-ADAP antiserum. In bone marrow cell suspensions, granulocytes (CD11b^+^ Ly6G^+^) were discriminated from macrophages (CD11b^+^ F4/80^+^). Both cell types showed clear reduction of ADAP expression in conditional k.o. mice compared with Cre control mice (Figure 3A). This deletion was also seen in splenic granulocytes. In splenic macrophages of conditional k.o. mice, two populations were detected: one population with ADAP expression comparable to control mice and one population with reduced ADAP expression. This observation indicates that the LysM-Cre promotes good deletion of ADAP in neutrophils, whereas the deletion in the splenic macrophage population is less efficient (Figure 3A). Microglia are the brain-resident macrophages and originate from hematopoietic progenitors. Therefore, we started to explore recombination efficacy of LysM-Cre in microglia. Leukocytes were isolated from the brain adult mice and analyzed by flow cytometry as CD45 low CD11b^+^ cell population (Figure 3B). Histograms depict ADAP expression in Cre control mice (ADAP^wt/wt^ LysM-Cre^het^) and reduction (but no loss) of ADAP expression in ADAP knockout mice (ADAP^fl/fl^ LysM-Cre^het^).

Next, we analyzed the frequency of granulocytes and macrophages in primary (bone marrow) and secondary lymphoid organs (spleen) of conditional k.o. mice and Cre control mice. The total numbers of cells in the bone marrow and the portion of macrophages and granulocytes in the bone marrow were identical in both genotypes (Figure 3C). The same result was achieved comparing the percentage of these myeloid cells in the spleen (macrophages and granulocytes) and in the peripheral blood (granulocytes; Figure 3D). DCs are not targeted by LysM-Cre mediated recombination (38). We did not observe differences in the proportion of CD11c+ cells between conditional k.o. mice and Cre control mice (Figures 3C,D).

CNS-resident microglia as well as invading macrophages and neutrophils contribute to the CNS autoimmune inflammation (39, 40). Based on our previous observation that conditional knockout mice with specific loss of ADAP in T cells and NK cells showed less severe EAE (Figures 1E, 2E), we induced the disease by active immunization with MOG_35−55_ peptide in conditional knockout mice with reduction of ADAP expression in myeloid cells and monitored the disease progression by clinical assessment. Mice expressing the wildtype allele in the presence of Cre recombinase (ADAP^wt/wt^ LysM-Cre^het^) exhibited a typical disease course. In contrast, conditional ADAP knockout mice (ADAP^fl/fl^ LysM-Cre^het^) developed a significantly milder disease showing fewer clinical symptoms (Figure 3E). These results indicate that expression of ADAP in myeloid cells partially contributes to the pathogenesis of EAE.

### Platelet-Specific Conditional ADAP Knockout Mice Develop Enhanced Active EAE

**Figure 4:**
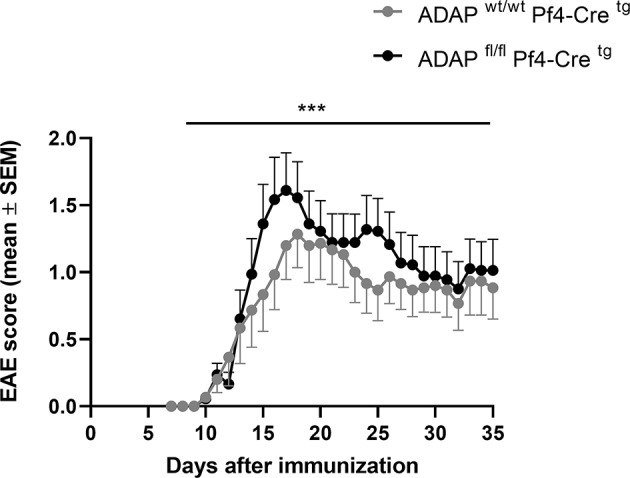
Active EAE in platelet-specific conditional ADAP knockout mice. EAE was induced following immunization with MOG_35−55_ peptide in CFA. The clinical score of EAE was assessed for 35 days after immunization (mean ± SEM; *n* = 15 for Cre control (ADAP^wt/wt^ PF4-Cre^tg^) and *n* = 18 for conditional ADAP knockout (ADAP^fl/fl^ PF4-Cre^tg^); ***P < 0.0001 conditional ADAP knockout vs. Cre control).

We generated platelet-specific ADAP knockout mice (ADAP^fl/fl^ PF4-Cre^tg^) and reported recently that these mice resemble the phenotype of conventional ADAP knockout mice showing thrombocytopenia and augmented re-bleeding from tail wounds. *In vitro*, ADAP-deficient platelets display impaired CLEC-2-mediated αIIbβ3 integrin activation, altered fibrinogen binding and reduced TGF-β1 and PF4 release (31). The role of platelets in EAE is controversially discussed (29, 41, 42). Furthermore, we could demonstrate that the loss of ADAP exclusively in platelets (ADAP^fl/fl^ PF4-Cre^tg^) caused a more severe disease course in the model of passive adoptive transfer EAE compared to that in Cre control mice (31). To allow a direct comparison of EAE course with the conditional ADAP knockout in T cells, NK cells and myeloid cells, here, we induced the active EAE by immunization of mice with MOG_35−55_ peptide also in the platelet-specific ADAP knockout mice. As shown in Figure 4, mice with the platelet-specific deletion of ADAP (ADAP^fl/fl^ PF4-Cre^tg^) developed a significantly more severe EAE compared to control animals (ADAP^wt/wt^ PF4-Cre^tg^). These data suggest a role of platelets in EAE that is at least partially controlled by ADAP.

**Figure 5:**
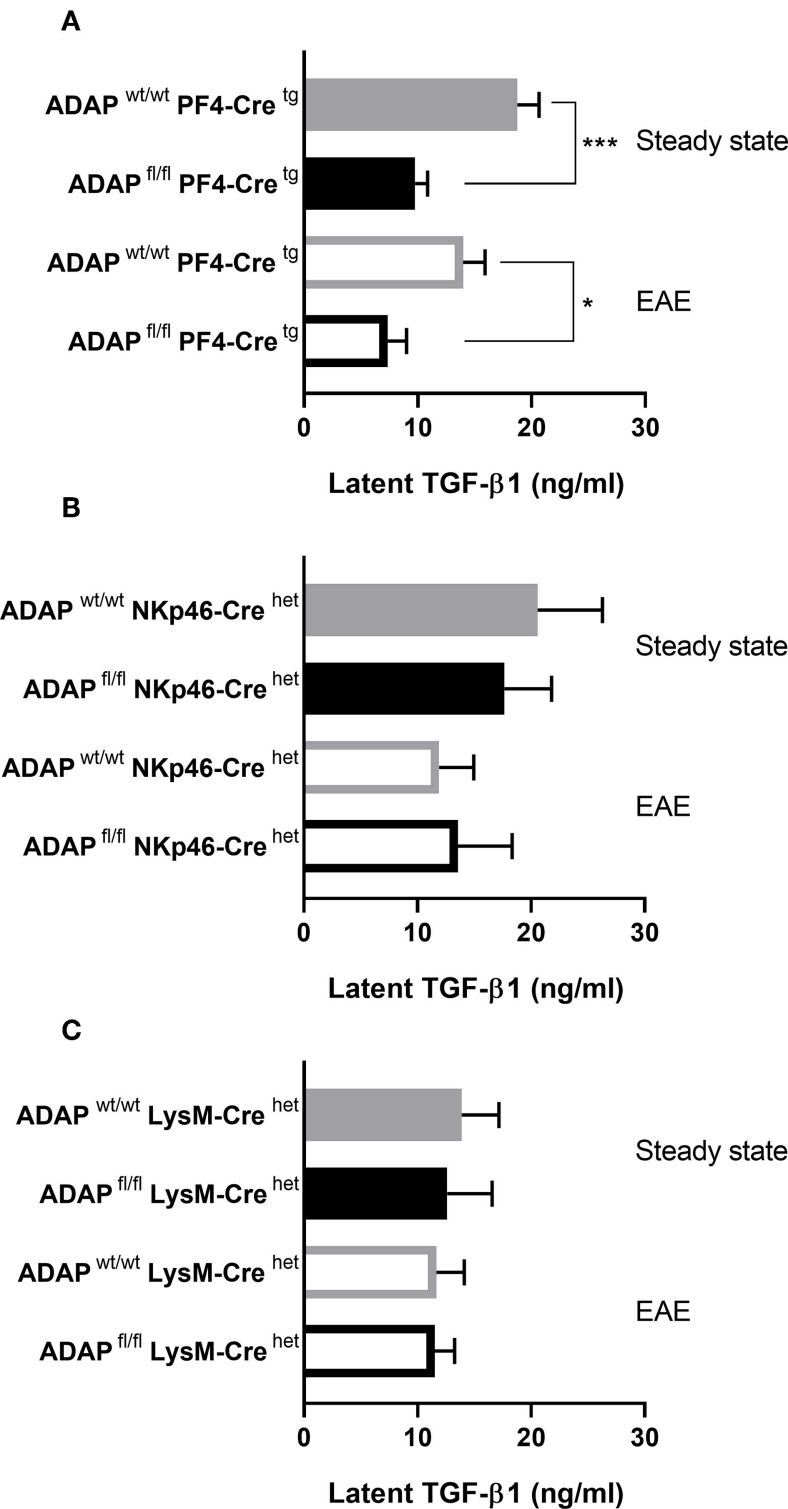
Concentration of latent TGF-β1 in conditional ADAP knockout mice. Concentration of latent TGF-β1 was measured in platelet-free plasma of platelet-specific **(A)**, NK cell-specific **(B)**, and myeloid cell-specific **(C)** ADAP knockout mice under physiological steady state condition (filled columns) and at day 35 of active EAE (empty columns). Concentrations are given as mean + SEM of at least seven individual mice. ***P < 0.001; *P < 0.05 conditional ADAP knockout vs. Cre control.

We could previously show that systemic injection of TGF-β1 reduced the EAE severity in the passive adoptive transfer model in platelet-specific ADAP knockout mice (31). Based on this observation, we wanted to compare the plasma TGF-β1 concentrations of the different conditional ADAP knockout mice. To this end, we analyzed the level of circulating latent TGF-β1 at steady state and at day 35 of active EAE. As shown in Figure 5A, platelet-specific ADAP knockout mice revealed significantly reduced levels of circulating latent TGF-β1 at steady state and at day 35 of active EAE compared to the respective control animals. In contrast, the concentrations of latent TGF-β1 were similar in NK cell-specific as well as in myeloid cell-specific conditional knockout mice and their respective control mice. In addition, there was no difference in the TGF-β1 concentration between the physiological steady state and during the autoimmune disease (Figures 5B,C). This result points toward a specific effect of ADAP depletion in platelets.

## Discussion

We have previously shown that conventional ADAP knockout mice develop a significantly milder clinical course of EAE by mainly T cell-independent mechanisms (20). To dissect the impact of different ADAP-expressing cell types on the ameliorated EAE severity, we generated conditional knockout mice. Our results show that the loss of ADAP in T cells, myeloid cells and NK cells resulted in less severe EAE whereas loss of ADAP in platelets leads to an exacerbated EAE compared to control animals.

Using Cre recombinase expressed as transgene under the control of the Lck promotor, we generated conditional knockout mice with deletion of ADAP in the T cell lineage. The major stages of T cell development in the thymus can be delineated by the expression of CD4 and CD8 which define the stages of double negative CD4^−^ CD8^−^ (DN), double positive CD4^+^ CD8^+^ (DP), and single positive CD4^+^ or CD8^+^ (SP) thymocytes. The Lck promotor is first expressed during early thymic development at the DN stage. DP thymocytes, the major population within the thymus, showed complete loss of ADAP protein in the ADAP^fl/fl^ Lck-Cre^tg^ mice. Unlike in thymocytes, peripheral splenic T cells exhibited a reduction in ADAP expression. This lower recombination rate in splenic T cell was reported earlier. It was suggested that rare thymocytes with incomplete deletion mature, leave the thymus and undergo clonal expansion in the periphery (43).

The distribution of thymocytes revealed the known abnormalities shown by Wu et al. (44): the higher percentage of DP cells and the lower proportions of SP cells. Interestingly, the conditional knockout mice did not exhibit the overall decreased thymic cellularity reported earlier (5, 6, 44). The presence (in conditional knockout) or absence (in conventional knockout) of ADAP in dendritic cells and macrophages that are involved in selection processes during thymocyte development might be an explanation for this observation. In the spleen, conditional and conventional ADAP knockout mice show the same phenotype of significantly reduced relative numbers of T cells (6, 44).

The NKp46-Cre knock-in mouse is widely used to delete genes containing flanking loxP sites in NK cells. Using these mice, we could demonstrate that the loss of ADAP in bone marrow NK cells and in splenic NK cells is almost complete. Cre expression is induced under the control of the NKp46 promotor at stage 3 of NK cell development (32). Our results showing normal maturation of ADAP-deficient NK cells in the bone marrow are therefore consistent with earlier published data (45). Consequently, the distribution of mature NK cells in spleen and in peripheral blood revealed no significant differences between the conditional knockout mice and Cre control mice.

Besides conventional NK cells, the existence of distinct tissue-specific NK cell populations is accepted (46). In contrast to conventional NK cells that develop in the bone marrow, evidence is accumulating that these organ-specific NK cells develop at extramedullary sites in the thymus, gut, liver, and uterus (47). We are aware of the problem that these cells express NKp46 and Cre-mediated recombination might occur. However, we assume that the impact of these tissue–resident NK cells on the disease model of EAE can be neglected.

Myeloid cells represent a heterogeneous population including mononuclear and polymorphonuclear phagocytes. Abram et al. (38) performed a detailed analysis of specificity and efficiency of 13 different myeloid-Cre mouse strains crossed to ROSA26-EYFP reporter mice and assessed YFP expression using a standard protocol. LysM-Cre mice show up to 70% deletion in neutrophils. In addition, LysM-Cre promotes deletion with high efficiency in alveolar macrophages (≈ 95%) and peritoneal macrophages (≈ 90%) and with lower efficiency in splenic macrophages (≈ 40%). For peripheral blood monocytes, the level of YFP^+^ cells is approximately 40% (38). Our results show a clear reduction of ADAP expression in granulocytes and bone marrow macrophages. In spleen macrophages, we detected two populations with high and low ADAP expression, respectively. Our results closely match the reported data from Abram et al. (38). However, this direct comparison is hampered by the different methods used: quantification of a fluorescent marker vs. staining with a specific antibody. The LysM promotor also targets microglia and showed an average recombination rate of 45% (48). Brain microglia cells of the ADAP^fl/fl^ LysM-Cre^het^ mice revealed a reduction of ADAP expression but no complete loss confirming the relatively low recombination efficiency. Our immunophenotyping data show that loss of ADAP expression in myeloid cells did not affect the frequency of these cells in the primary and secondary lymphoid organs of ADAP^fl/fl^ LysM-Cre^het^ mice.

Only recently, we provided evidence that the PF4-Cre mediated deletion of ADAP is highly efficient and specific. The ADAP^fl/fl^ PF4-Cre^tg^ mice resemble the phenotype of conventional ADAP knockout mice showing thrombocytopenia and augmented re-bleeding from tail wounds. The loss of ADAP in the megakaryocytic lineage had no impact on immune cell distribution in the lymphoid organs (31).

Overall, these mouse strains show deletion or reduction of ADAP expression in the specific target cell population. So far, no other reports using conditional ADAP knockout mice are available except for platelets (31, 49). The characterization of the conditional knockout mice revealed unaltered development and distribution of immune cells in the primary and secondary lymphoid organs when ADAP was deleted in myeloid cells, NK cells and platelets. Ablation of ADAP in T cells resembled the T cell phenotype of conventional ADAP knockout mouse. We are aware that inflammatory conditions like EAE could induce changes in Cre-mediated deletion efficiency and specificity compared to steady state. At least for PF4-Cre mediated recombination we could exclude this possibility during *in vitro* activation (31).

We used these characterized conditional knockout strains and performed comparative EAE experiments. To investigate the role of ADAP expressing immune cells during early and late stages of the autoimmune disease, we induced active EAE by immunization with the immunogenic MOG_35−55_ peptide in CFA.

In our previous study, we provided evidence that the resistance of conventional ADAP knockout mice to EAE is partially caused by T cell-independent mechanisms. Here, we show that mice with ADAP deficiency restricted to T cells developed less severe EAE. The results of these two studies seem contradictory. However, the impaired function of ADAP-deficient T cells (proliferation, IL-2 production, adhesion, NF-κB activation) had previously been characterized in detail (5–7). Thus, the reduced EAE severity in T cell-specific ADAP knockout mice is not surprising. In addition, induction of EAE after adoptive transfer of ADAP-deficient T cells into T cell-deficient mice caused a clear delay in disease onset compared to the adoptive transfer of ADAP-sufficient T cells (20). This observation indicates a partial T cell-dependent role of ADAP in EAE. Analysis of the different conditional ADAP-knockout strains allowed us to draw a more precise conclusion: T cell-dependent and T cell-independent mechanism are responsible for reduced EAE severity in conventional ADAP knockout mice. Concerning the T cell-dependent mechanism, two aspects have to be mentioned. First, the reduced numbers of mature T cells, especially CD8^+^ T cells, in the periphery of conditional knockout mice. Second, the well-known impaired T cell function in the absence of ADAP. At present, we cannot discriminate between these two possibilities. We assume that both aspects contribute to the reduced EAE severity in the T cell-specific ADAP knockout mouse. Conditional ablation of ADAP separately in either CD4^+^ or CD8^+^ T cells would help to clarify this question.

In this study, we provide evidence that ADAP deficiency restricted to NK cells reduced EAE severity. We hypothesize that primarily the disturbed IFN-γ production by NK cells causes the better outcome of EAE in NK cell-specific conditional ADAP knockout mice. Rajasekaran et al. (11) demonstrated that cytokine production but not cytotoxicity of NK cells is regulated by ADAP. Therefore, we rule out the cytotoxic function of ADAP-deficient NK cells as a factor affecting reduced EAE severity. Besides, we suggest that potential adhesion and migration defects of ADAP-deficient NK cells should be taken into account.

Previous studies reported that the ILC3 population (innate lymphoid cells type 3) in the meninges is involved in the pathogenesis of EAE by sustaining the neuroinflammation (50). These cells express NKp46, the transcription factor RORγt, and produce the cytokines IL-17 and GM-CSF. We cannot exclude the possibility that in this minor subpopulation in the meninges Cre-mediated deletion of ADAP occurs and therefore enhances the effect of milder EAE severity in ADAP^fl/fl^ NKp46-Cre^het^ mice.

In conditional knockout mice with specific deletion of ADAP in myeloid cells, we observed an attenuated disease course compared to control mice. Neutrophils contribute to the pathogenesis of EAE by different mechanisms including release of cytokines, proteases, reactive oxygen species, phagocytosis and interaction with other immune cells [reviewed in (51)]. To access the CNS, circulating neutrophils have to leave the vasculature and to migrate to sites of neuroinflammation. Deficiency in ADAP attenuates E-selectin-mediated slow leukocyte rolling and adhesion of neutrophils and protects mice from acute kidney ischemia-reperfusion injury (13). This adhesion defect might explain our observation of lower EAE severity when ADAP is deleted in myeloid cells. It is difficult to estimate the impact of ADAP in monocytes and macrophages in the EAE model. The recombination efficiency is low and varies depending on the tissue localization of the macrophages. In our previous study, lethally irradiated ADAP knockout recipient mice reconstituted with wildtype bone marrow developed milder EAE compared with wildtype recipients (20). We concluded that radio-resistant cells could be partially responsible for the attenuated EAE severity. Based on experimental data of normal function in the absence of ADAP we excluded a role of microglia. Therefore, the possibility that other ADAP-deficient radio-resistant myeloid cells might be responsible for the attenuated EAE severity still exists. Mast cells are candidates that fulfill these criteria and will be in focus of our future research.

Recently, we reported the unexpected finding that mice with platelet-specific ablation of ADAP exhibited enhanced EAE severity in the passive EAE model where *ex vivo* activated myelin-specific TCR transgenic T cells (2D2 T cells) were adoptively transferred. In addition, we provided evidence that thrombocytopenia and reduced concentration of TGF-β1 are responsible for the more severe course of EAE. Within the present study, we induced active EAE. In active EAE, both the induction and effector phase of the disease take place *in vivo*. We confirmed our previous data (31) showing augmented disease severity in mice lacking ADAP specifically in platelets. The difference in the clinical course of EAE was especially pronounced during the early phase of the disease (at days 10–15). This result implicates a regulatory role of ADAP in platelets during induction of EAE. We hypothesize that also in active EAE the reduced level of TGF-β1 in the platelet-specific conditional knockout mouse is responsible for enhanced disease severity. *In vivo* administration of TGF-β1 improved EAE severity in the model of passive EAE in mice lacking ADAP in platelets. However, this effect was only achieved in a preventive but not in a therapeutic approach (31). This result indirectly supports our hypothesis that platelet-derived ADAP plays an important suppressive role during the induction phase of EAE. Platelets are the most important source of TGF-β1. As a potent anti-inflammatory cytokine, TGF-β1 is required to convert conventional CD4^+^ T cells into induced regulatory T cells (52). The reduced TGF-β1 concentration thus might impair the development of regulatory T cells *in vivo*, leading to reduced immune suppression and enhanced EAE severity.

The question remains about the interplay between the described cell type-specific effects on the EAE course in conventional ADAP knockout mice. We believe that the impact of a single cell type is determined by the significance of this cell population for the pathogenesis of EAE. Most importantly, autoreactive T cells primed by (myeloid) antigen presenting cells are critical for this autoimmune disease. Infiltrating NK cells and platelets, although contributing, are less important. Since loss of ADAP in T cells, myeloid cells, and NK cells reduced EAE severity, the additive effects of these cells overbalances the EAE enhancing effect of ADAP-deficient platelets.

Summarizing, we can conclude that in conventional ADAP knockout mice T cell-dependent and T cell-independent mechanisms are involved in the resistance to EAE. Not a single cell type but different ADAP expressing myeloid cells as well as NK cells might exert synergistic effects contributing to significantly milder EAE. These investigations expand our knowledge about the complex role of the adapter protein ADAP in immune cells.

## Data Availability Statement

All datasets generated for this study are included in the manuscript/Supplementary Files.

## Ethics Statement

This study was carried out in accordance with the recommendations of the German Tierschutzgesetz. The protocol was approved by Landesverwaltungsamt Sachsen-Anhalt, Referat 405 (42502-2-1273 Uni MD).

## Author Contributions

JR and AR designed and performed the research and analyzed data. KG, CM, and MV performed experiments and analyzed data. AR wrote the manuscript. SK, DR, and BS contributed to interpretation of data, critical writing, and revising the manuscript.

### Conflict of Interest

The authors declare that the research was conducted in the absence of any commercial or financial relationships that could be construed as a potential conflict of interest.
