# Supplementary material for: Immune Cell-Type Specific Ablation of Adapter Protein ADAP Differentially Modulates EAE
Source: Front Immunol. 2019 Oct 1;10:2343. doi: 10.3389/fimmu.2019.02343 (PMC6779796; doi:10.3389/fimmu.2019.02343)
Supplement: Supplementary file 1 [file Data_Sheet_1.docx]

**Supplementary Material**

**
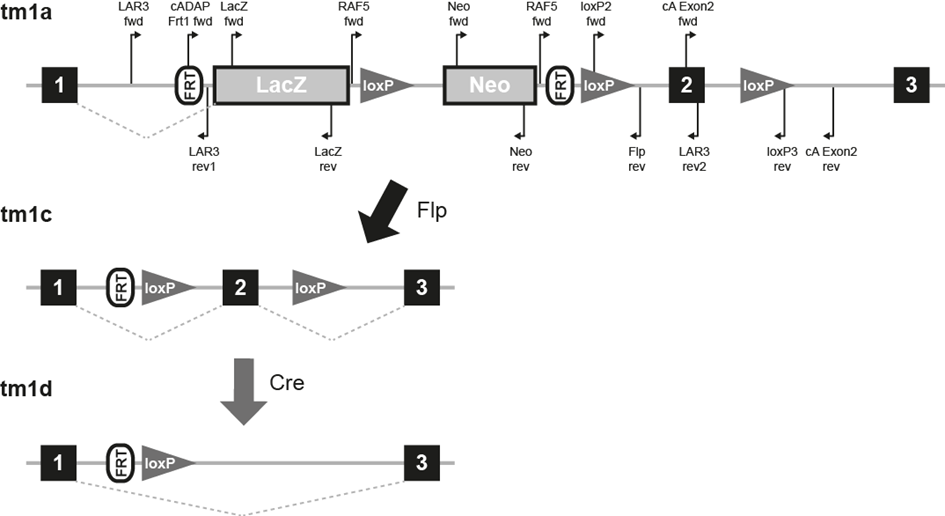
**

**Figure S1. Generation of conditional knockout mice.**

Scheme of generation of conditional knock-out mice based on mice containing the
knock-out first allel­­e C57BL/6N-Fyb^tm1a(EUCOMM)Hmgu^/Cnrm (EUCOMM) (tm1a). LacZ and neomycin-resistance (Neo) cassettes (light gray boxes) were both removed by breeding with transgenic mice expressing the Flp recombinase resulting in floxed alleles (containing *loxP* sites) and restoring the wildtype (tm1c). To generate mice with the deletion of ADAP exclusively in different lineages (tm1d), the mice with floxed alleles (tm1c) were crossed with mice carrying the Cre recombinase under control of a lineage-specific promoter. Black boxes labelled with "1", "2" and "3" represent the exons 1, 2 and 3 of the mouse ADAP (Fyb) locus. Location and direction of primer sequences are indicated below or above the knock-out first allele (tm1a).

**Table S1. Primer sequences used for genotyping**

| **PCR** | **Primer** | **Sequence (5´🡪 3´)** |
| --- | --- | --- |
| **LacZ** | LacZ fwd | cta cac caa cgt gac cta tcc |
|  | LacZ rev | ctc atc cat gac ctg acc at |
| **Neo** | Neo fwd | gat gcc gcc gtg ttc c |
|  | Neo rev | gcc cct gat gct ctt cgt c |
| **Flp** | cADAP Frt1 fwd | ggc gcc gga acc gaa gtt |
|  | Flp rev | ccc cag acc tag cac tca ca |
| **Con ADAP** | cA Exon2 fwd | aaa cat caa acc tcc cct tga c |
|  | cA Exon2 rev | tca cga ggt gag atc gtc tg |
|  | cA Loxp3(a) rev(2) | ggc gag ctc aga cca taa ctt cg |
| **LAR3** | LAR3 fwd | ggg agg tgg gaa tat ggg tg |
|  | LAR3 rev1 | cac aac ggg ttc ttc tgt tag tcc |
|  | LAR3 rev2 | ggt caa ggg gag gtt tga tgt |
| **RAF** | RAF5 fwd | cac acc tcc ccc tga acc tga aac |
|  | loxp2 fwd | tac att ata cga agt tat gtc gag |
|  | Flp rev | ccc cag acc tag cac tca ca |
| **PF4-Cre** | Cre fwd (PF4) | ccc ata cag cac acc ttt tg |
|  | Cre rev (PF4) | tgc aca gtc agc agg tt |
| **Lck-Cre** | Lck Cre for | cct tgg tgg agg agg gtg gaa tga a |
|  | Lck Cre rev | aat gtt gct gga tag ttt tta ctg c |
| **NKp46-iCre** | NKp46ex7 for | gga act gaa ggc aac tcc tg |
|  | NKp46ex7 rev | ttc ccg gca aca taa aat aaa |
|  | NKp46IRES rev | ccc tag gaa tgc tcg tca ag |
| **LysM-Cre** | MLYS1 | ctt ggg ctg cca gaa ttt ctc |
|  | MLYS2 | tta cag tcg gcc agg ctg ac |
|  | CRE8 | ccc aga aat gcc aga tta cg |
